# Clinical nephrology research in low-resource settings: opportunities, priorities, and challenges for young investigators - Proceedings from the 10^th^ Conference on Kidney Disease in Disadvantaged Populations in Cape Town, South Africa, March 2015

**DOI:** 10.5414/CNP86S110

**Published:** 2016-07-29

**Authors:** Shuchi Anand, John W. Stanifer, Bernadette Thomas

**Affiliations:** 1 Division of Nephrology, Department of Medicine, Stanford University School of Medicine, Palo Alto, CA,; 2 Division of Nephrology, Department of Medicine, Duke University,; 3 Duke Global Health Institute, Duke University, Durham, NC, and; 4 Division of Nephrology, Department of Medicine, University of Washington, Seattle, WA, USA; *All authors contributed equally to this manuscript.

**Keywords:** non-communicable disease, young investigators, low-resource settings, global health, research priorities

## Abstract

The increased recognition of the growing, worldwide burden of kidney disease has led to calls for prioritizing nephrology research in a global context. However, many challenges exist for young investigators interested in studying kidney disease in low-resource global settings. A lack of clear research priorities, limited funding options, poor infrastructure, difficulty forming partnerships, and unestablished paths for career advancement are a few examples. To discuss these issues, we held a moderated panel discussion in March 2015 as part of the 10^th^ Conference on Kidney Disease in Disadvantaged Populations in Cape Town, South Africa. A group of senior investigators discussed research priorities for studying kidney disease in a global context, collaborations for clinical research, and strategies for dealing with the unique challenges faced by young investigators working in this field.

## Introduction 

Noncommunicable diseases (NCDs) disproportionately affect the economic, social, and health outcomes of lower- and middle-income countries (LMIC) [1]. Kidney disease is gaining recognition among experts and policy makers as an NCD with significant morbidity and mortality, and there are increasing calls for prioritizing nephrology research in a global context [2, 3, 4]. Young investigators are beginning to address this need, and they are positively influencing this emerging field of study [5, 6, 7, 8, 9]. However, many challenges exist for young investigators, and no road map exists for addressing these challenges. 

**Figure 1.**
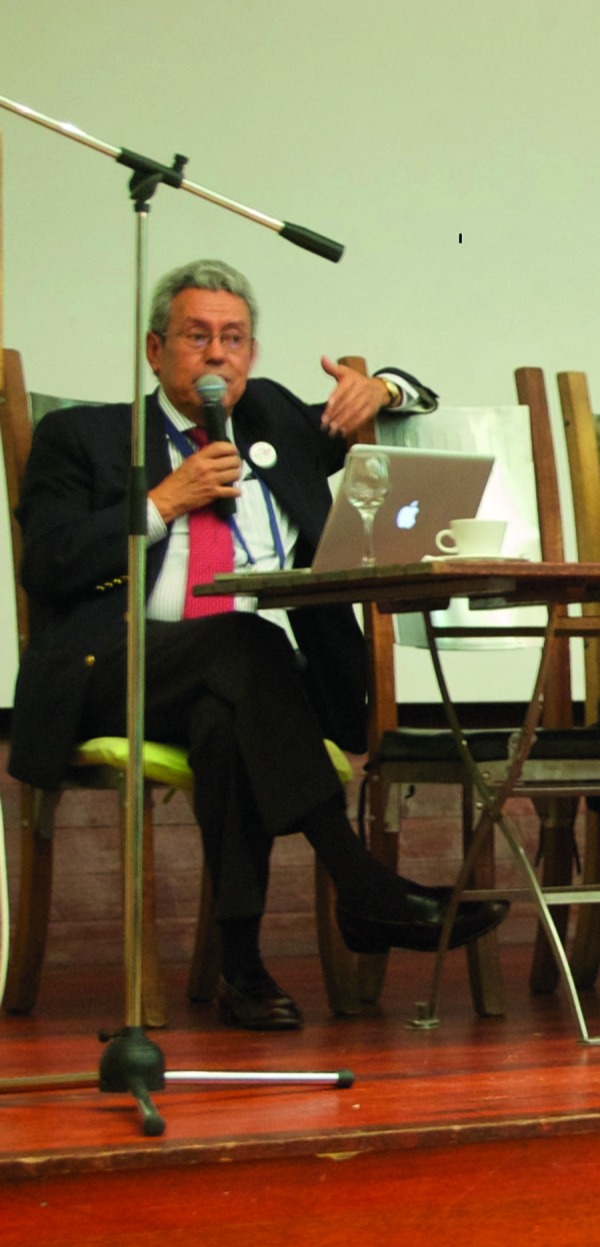
Dr. Guillermo Garcia-Garcia discussing the importance of using epidemiological data to efficiently focus local research efforts.

**Figure 2.**
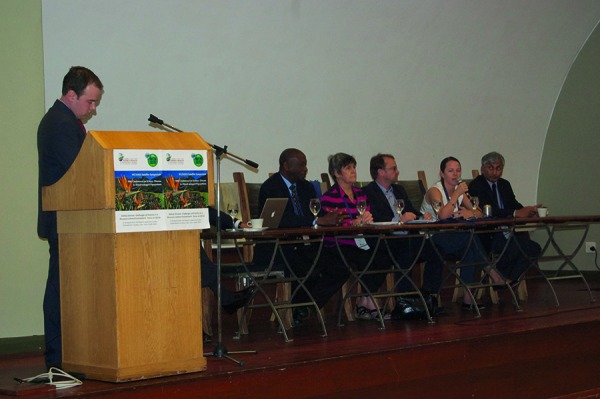
Moderated panel of senior investigators (shown from left to right: Drs. John Stanifer (moderator), Lawrence Agodoa, Wendy Hoy, Roberto Picoits-Filho, Karen Yeates, and Vivek Jha).

To this end, the 10^th^ Conference on Kidney Disease in Disadvantaged Populations was held on March 17 – 18^th^, 2015in Cape Town, South Africa as a satellite symposium of the International Society of Nephrology (ISN) World Congress of Nephrology 2015. As part of the symposium, a moderated panel discussion on career development for young investigators researching kidney disease in low-resource settings was held on March 17^th^. The panel was composed of a group of senior investigators including Drs. Guillermo Garcia-Garcia (Figure 1), Lawrence Agodoa, Wendy Hoy, Roberto Picoits-Filho, Karen Yeates, and Vivek Jha. The session was moderated by Drs. Shuchi Anand, Bernadette Thomas, and John Stanifer (Figure 2). The discussion centered around three key topics: research priorities, building collaborations for clinical research, and career challenges faced by young investigators. 

## How should we frame nephrology research questions when working in low-resource settings? 

From the moderated discussion, several topics emerged as important considerations for effective research in low-resource global settings. These topics were considered especially pertinent for young investigators. 

### Create research questions of broad interest 

Young investigators should be prepared to frame their research goals in a way that can answer questions of significance both locally and more broadly (e.g., at the country or regional level). While most research begins with a very specific question in mind, given the paucity of data (and resources), successful investigators working in low-resource settings must be able to situate their research within the broader context. This underscores that even small projects in a local population can take on greater significance. 

As an example, the ISN’s 0x25 Campaign, which seeks to address the global burden of acute kidney injury. Similar to HIV, acute kidney injury most often affects the young and economically-productive members of society in LMICs [9, 10]. As such, preventing and treating it at a local level requires engaging with the regional and global health, economic, and social equity issues. Therefore, even local projects that aim to tackle a specific aspect of acute kidney injury in a specific population can be linked to the broader effort of addressing acute kidney injury as a health priority in LMICs. 

Research agendas are not mutually exclusive. Global health nephrology encompasses many facets that include epidemiology, ethics, chronic kidney disease detection and treatment, and delivery of renal replacement therapies including transplant. Research across these areas can be complementary. For example, developing a renal registry to gather information on the epidemiology of chronic kidney disease in a region would be informative in formulating policy for the delivery of renal replacement therapies or in implementing preventative strategies targeting multiple noncommunicable diseases. 

### Prioritize the collection of epidemiological data and registries 

Epidemiological data for acute and chronic kidney disease are sparse in most LMICs [11, 12, 13]. We need high-quality epidemiology studies from these regions in order to demonstrate the extent of the burden. This will in turn bring increased visibility to important issues in low-resource settings and can help focus multi-national efforts in priority setting, funding agendas, and collaboration. 

Increasing our epidemiological knowledge base will also further our understanding of local problems. Some of these issues will be common across regions, and some will be unique to the local settings, but understanding these challenges within a population will highlight local disparities. Given the limited funding opportunities and the challenges of conducting research in low- and middle-income countries, using epidemiological data to efficiently focus research efforts on the most critical local problems with high disparities but broad implications will be of high value and importance. 

### Align global interests with local needs 

Young investigators need to be innovative in aligning global priorities with local needs. Health challenges in low-income countries extend well beyond the field of nephrology, but investigators can incorporate nephrology research within other high-profile, active areas of research and public health attention. For example, chronic kidney disease is etiologically related to communicable and non-communicable diseases as well a multitude of environmental factors [14]. As such, it can be studied from the perspective of cardiovascular and cerebrovascular disease and linked to NCD management and outcomes, which are increasingly being recognized as a global threat [2, 15]. On the other hand, it can also be studied in the context of communicable diseases, such as HIV, malaria, and tuberculosis, which have large and stable funding sources and are linked to higher risks of acute and chronic kidney disease. Likewise, kidney disease can be studied in the context of maternal mortality especially as it pertains to pre-eclampsia and eclampsia, and it can be studied in the context of high-risk environmental and geographic profiles such as hazardous mining operations, water and food chain contamination, and poor urban planning. 

Answering questions about healthcare economics and health services delivery may also provide a particularly attractive way to frame global health nephrology research. Governments and healthcare systems are constantly pressured to reduce costs and increase efficiency; therefore, any research that incorporates these objectives could align global or regional priorities with local needs. 

Finally, young investigators should search for ways to apply research findings from LMICs to high-income countries: the so-called reverse innovation phenomenon. Even high-income countries have disenfranchised populations with disproportionately poor outcomes. Demonstrating novel ways to address disparities in low-income countries could be applicable to multiple countries regardless of income level. Another area that could produce reverse-innovation, while at the same time answering questions about healthcare economics, is the delivery and mechanisms of renal replacement therapies. If renal replacement therapies are to be available in low-income settings, then new technologies will need to be created, and resourceful modes of delivery will need to be developed. In high-income countries like the United States where dialysis costs consume increasingly large proportions of the healthcare budget, this type of research could also be of high value [16]. 

### How should we form collaborations for implementing clinical research in low-resource settings? 

The panel discussed approaches to forming collaborations that were of particular relevance for young investigators. Following the model of communicable disease, most research from LMICs that focuses on NCDs, e.g., the International Polycap study 3 (TIPS3), has involved partnerships with researchers from high-incomes countries, and often times, the researchers in these partnerships have distinct roles. Lack of research expertise in LMICs may in part drive this division of roles, but frequently a lack of infrastructure in the LMIC partner-country necessitates a ground-up approach. 

However, for young investigators, such an approach can be a significant barrier as it often requires training local personnel, building a supply chain, establishing financial networks, and navigating bureaucracies [17]. The panel discussed the strategic value of a central consortium that could house information about ongoing studies, help form partnerships between young investigators, and allow for setting-specific consultation regarding data management and study implementation. For example, the H3AFRICA Study has established a multi-country research network that has facilitated additional projects for young investigators through its established infrastructure, capacity, and resources [18]. 

Supporting such efforts could transform the traditional partnership roles between researchers from high-income countries and LMICs. Currently, methodological expertise and grant funding typically come from the high-income partner, but a central consortium could emphasize transfer of several responsibilities to the LMIC partner such as data collection, data management, and implementation. If skills-transfer is a focus of collaborative partnerships, then such redistribution of roles would reduce many barriers and establish an infrastructure facilitating the development of larger research agendas by LMIC partners. 

Finally, partnerships must be built on mutual respect, congeniality, equity, and common interest. The panel suggested many additional characteristics that were considered critical for building successful partnerships between investigators in high-income and LMICs (Table 1). 

### What are the career and personal challenges faced by young investigators pursuing nephrology research in low-resource settings? 

Challenges for young investigators specific to clinical research in low-resource settings include significant time investment, lack of partnerships, language differences, and limited infrastructure and ancillary support. Additionally, the panelists acknowledged the dearth of research funding sources and the opportunity costs in terms of career advancement. 

These challenges mean that young investigators must not only have a strong passion for their research, but they must also obtain high-value results for low cost, build overlapping infrastructure support, leverage technology to more efficiently facilitate communications and networking, and have an in-depth understanding of the local health system and needs. The panelists also stressed that while young investigators should leverage technology to more efficiently facilitate communications, direct time in the local setting is critical to successful implementation. As an example, opportunities such as the Fogarty International Scholarship provide a means for protected researched time which can in turn allow for much-needed direct contact. 

Young investigators will also need institutional support that recognizes the laborious nature of clinical research in low-resource settings, and academic institutions need to establish different promotion benchmarks for investigators working primarily in low-resource settings. 

## Conclusions 

The increasing recognition of kidney disease as an important research topic worldwide is leading to many unique opportunities for young investigators; however, many challenges exist, including limited funding options, unclear research priorities, lack of infrastructure, difficulty forming partnerships, and unestablished paths for career advancement. Young investigators must be creative in framing research questions that are of broad interest, prioritizing the collection and registration of epidemiological data, and be innovative in aligning global priorities with local needs. Forming collaborations is a critical element of successful clinical research in low-resource settings, and they should be built on mutual respect, congeniality, equity, and common interest. Finally, increased home institutional support with custom benchmarks for global health career pathways is much needed. 

## Acknowledgments 

Dr. Stanifer is supported by a National Institutes of Health (NIH) Research Training Grant (#R25 TW009337) funded by the Fogarty International Center and the National Institute of Mental Health. Dr. Anand is supported by NIH National Institute for Diabetes and Digestive and Kidney Health K23 (# DK101826-01). 

**Table 1.** Suggested characteristics for building successful partnerships between investigators in high-income and low- and middle-income countries (LMIC).

Suggested characteristics for partners from high-income countries:
Work locally to assess needs and knowledge gaps
Establish a two-way knowledge and skills transfer
Design studies that address sustainability and recognize local resources and constraints
Design studies that incorporate cultural differences
Ensure sensitivity to ethical concerns particularly when designing studies that involve testing an intervention or device
Understand that research should be a long-term investments aimed at improving outcomes
Suggested characteristics for partners from LMICs:
Be receptive to knowledge and skill transfers
Ensure the local relevance and applicability of the research
Advocate for long-term sustainability on a policy level
Search for funding opportunities
Disseminate results through publications and networking with local policymakers
